# The Expression of Anti-Müllerian Hormone Type II Receptor (AMHRII) in Non-Gynecological Solid Tumors Offers Potential for Broad Therapeutic Intervention in Cancer

**DOI:** 10.3390/biology10040305

**Published:** 2021-04-07

**Authors:** Jean-Marc Barret, André Nicolas, Anne Jarry, Olivier Dubreuil, Didier Meseure, Tilda Passat, Emeline Perrial, Cécile Deleine, Gabriel Champenois, Solenne Gaillard, Emilie Duchalais, Isabelle Ray-Coquard, Mehdi Lahmar, Charles Dumontet, Jaafar Bennouna, Céline Bossard, Sergio Roman-Roman, Jean-François Prost

**Affiliations:** 1 GamaMabs Pharma, Centre Pierre Potier, F-31100 Toulouse, France; odubreuil@gamamabs.fr (O.D.); gaillard.solenne@gmail.com (S.G.); mehdilahmar@yahoo.com (M.L.); jfprost@gamamabs.fr (J.-F.P.); 2 Department of Diagnostic and Theranostic Medicine, Platform of Experimental Pathology, Institut Curie, PSL Research University, F-75005 Paris, France; andre.nicolas@curie.fr (A.N.); didier.meseure@curie.fr (D.M.); gabriel.champenois@curie.fr (G.C.); 3 Université de Nantes, Inserm U1232, CRCINA, F-44000 Nantes, France; anne.jarry@univ-nantes.fr (A.J.); tilda.passat@etu.univ-nantes.fr (T.P.); cecile.deleine@univ-nantes.fr (C.D.); 4 Cancer Research Centre de Lyon, Inserm 1052, CNRS 5286, F-69008 Lyon, France; emeline.cros@univ-lyon1.fr (E.P.); charles.dumontet@chu-lyon.fr (C.D.); 5 Service de Chirurgie Digestive et Endocrinienne, Centre Hospitalier Universitaire Hôtel Dieu, F-44000 Nantes, France; emilie.dassoneville@chu-nantes.fr; 6 Laboratory RESHAPE U1290, Léon Bérard Cancer Centre, F-69008 Lyon, France; isabelle.ray-coquard@lyon.unicancer.fr; 7 Service Oncologie Digestive, Institut des Maladies de l’Appareil Digestif, Centre Hospitalier Universitaire Hôtel Dieu, F-44000 Nantes, France; jaafar.bennouna@chu-nantes.fr; 8 Service Anatomie Pathologique, Centre Hospitalier Universitaire Hôtel Dieu, Inserm 1232, CRCINA, F-44000 Nantes, France; celine.bossard@chu-nantes.fr; 9 Translational Research Department, Institut Curie, Research University, F-75005 Paris, France; sergio.roman-roman@curie.fr

**Keywords:** AMHRII, oncofetal antigen, colorectal cancer, protein expression, murlentamab

## Abstract

**Simple Summary:**

Until now, only a few studies have examined the AMHRII expression in tumors. Here, with more than 1000 tumor samples and using several complementary techniques we confirmed AMHRII expression in gynecological cancer and demonstrated AMHRII expression in certain non-gynecological cancers such as colorectal cancers. These findings open the way for new therapeutic approaches targeting AMHRII and emphasize the need to better understand the role of AMH/AMHRII in cancer.

**Abstract:**

The anti-Müllerian hormone (AMH) belongs to the TGF-β family and plays a key role during fetal sexual development. Various reports have described the expression of AMH type II receptor (AMHRII) in human gynecological cancers including ovarian tumors. According to qRT-PCR results confirmed by specific In-Situ Hybridization (ISH) experiments, AMHRII mRNA is expressed in an extremely restricted number of normal tissues. By performing ISH on tissue microarray of solid tumor samples AMHRII mRNA was unexpectedly detected in several non-gynecological primary cancers including lung, breast, head and neck, and colorectal cancers. AMHRII protein expression, evaluated by immunohistochemistry (IHC) was detected in approximately 70% of epithelial ovarian cancers. Using the same IHC protocol on more than 900 frozen samples covering 18 different cancer types we detected AMHRII expression in more than 50% of hepato-carcinomas, colorectal, lung, and renal cancer samples. AMHRII expression was not observed in neuroendocrine lung tumor samples nor in non-Hodgkin lymphoma samples. Complementary analyses by immunofluorescence and flow cytometry confirmed the detection of AMHRII on a panel of ovarian and colorectal cancers displaying comparable expression levels with mean values of 39,000 and 50,000 AMHRII receptors per cell, respectively. Overall, our results suggest that this embryonic receptor could be a suitable target for treating AMHRII-expressing tumors with an anti-AMHRII selective agent such as murlentamab, also named 3C23K or GM102. This potential therapeutic intervention was confirmed in vivo by showing antitumor activity of murlentamab against AMHRII-expressing colorectal cancer and hepatocarcinoma Patient-Derived tumor Xenografts (PDX) models.

## 1. Introduction

The Anti-Müllerian hormone type II receptor (AMHRII), also known as MIS type II receptor (MISRII or MISIIR), is a member of the transforming growth factor beta (TGF-β) receptor superfamily and was discovered in 1994 by two independent teams [1,2]. AMHRII specifically binds the anti-Müllerian hormone (AMH or Müllerian inhibiting substance or MIS for Müllerian inhibiting substance) [3] and plays a major role in male fetus sexual differentiation by inducing the regression of Müllerian ducts, precursors of female reproductive organs (uterus, fallopian tubes, and upper vagina) [4]. In adults, AMHRII displays a restricted expression profile with expression limited to granulosa cells in women from birth to menopause and acts as a modulator of follicular growth and cycling [5]. In adult males, AMHRII has been described to be expressed in Sertoli and Leydig cells and is involved in the regulation of androgen biosynthesis [6]; Inactivating mutations of AMHRII result in Leydig cell hyperplasia [7]. AMHRII expression has been also described in the brain but its role (neurogenesis, neuroprotection, and/or regulator of sex-linked bias) [8] remains unclear. More recently, AMHRII was detected in GnRH neurons suggesting a regulating role in the hypothalamic-pituitary-gonadal axis [9]. However, mutated forms of AMHRII do not appear to be associated with a neurological or behavioral impact [10].

Based on the observation of Professor R.E. Scully that Epithelial Ovarian Cancers (EOC) recapitulate embryonic Müllerian histology [11], it was hypothesized that these cancers could express AMHRII and be inhibited by anti-AMH treatment [12]. Several studies have confirmed the expression of AMHRII in gynecological cancer tissues [13,14,15,16,17] and pro-apoptotic/growth inhibition activity of AMH was repeatedly described in ovarian, cervical, and endometrial cancer cells, confirming the potential of AMHRII as a target for anticancer agents [18,19]. These observations led to the development of murlentamab, also named GM102, a humanized glyco-engineered anti-AMHRII monoclonal antibody. Following an extensive pharmacological profile analysis as well as toxicological studies in cynomolgus monkeys [20,21,22], a Phase 1 study of murlentamab has been initiated in patients with gynecological cancers (NCT02978755).

Due to the embryonic nature of the receptor, and the potential involvement of AMHRII in epithelial-mesenchymal transition in non-small cell lung cancer [23], a process common to solid tumors, we proceeded with an extensive investigation of AMHRII expression using complementary techniques in a variety of solid tumors. We observed expression of AMHRII in several non-gynecological cancers such as hepato-carcinomas (HCC), renal cell carcinomas (RCC), colorectal cancers (CRC), and non-small cell lung cancers (NSCLC). These results support potential indications for treatments with anti-AMHRII agents such as murlentamab. Moreover, preliminary studies with murlentamab showed antitumor activity in two AMHRII-expressing Patient-Derived tumor Xenograft (PDX) models, obtained from HCC and CRC samples, confirmed novel clinical perspectives for this agent.

## 2. Results

### 2.1. AMHRII Transcription Is Limited to Very Few Normal Tissue Types

**Figure 1:**
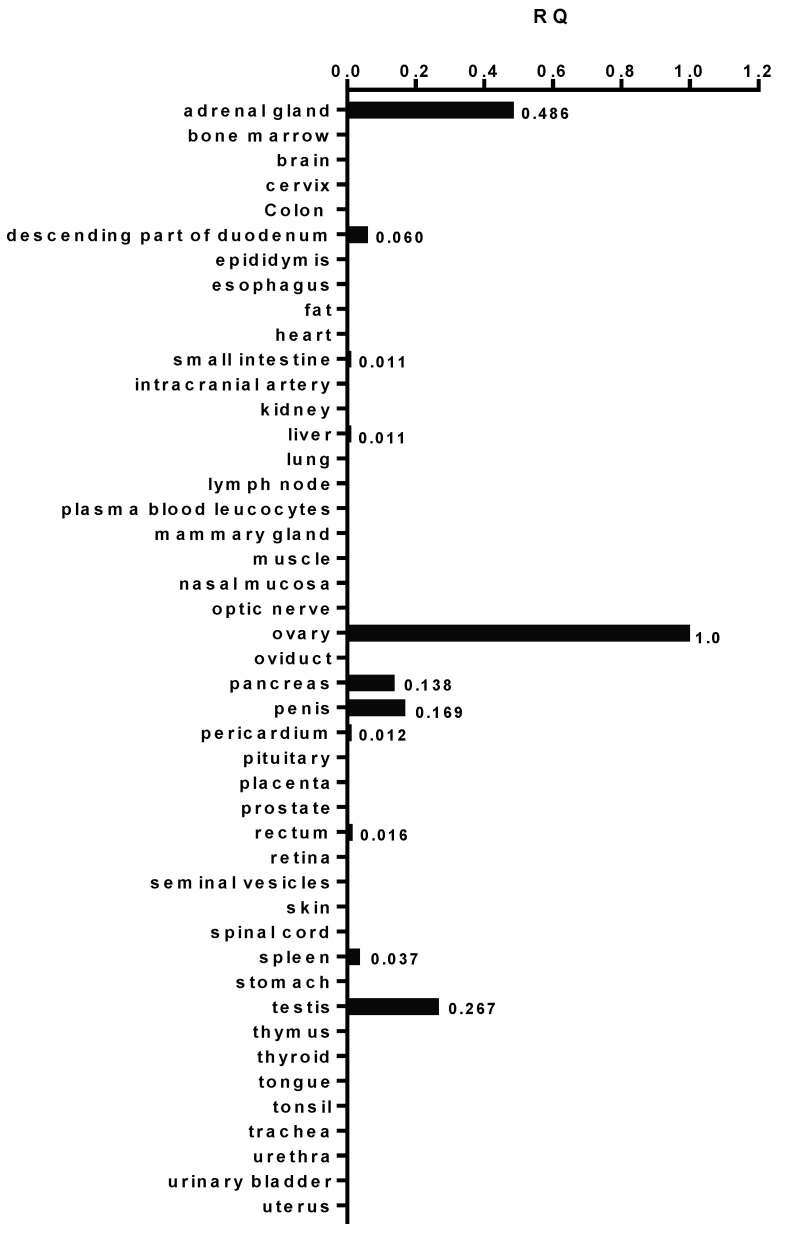
AMHRII expression in normal human tissues determined by RT-qPCR. Relative Quantification (RQ) values were calculated by using the delta Ct method.

Previous studies have reported that AMHRII was mainly expressed in the ovary [5] and testis [6] of human adults. Data from the Human Protein Atlas identified AMHRII RNA in only a limited number of organs: ovary, testis, adrenal gland and, to a lesser extent, pancreas and spleen (https://www.proteinatlas.org/ENSG00000135409-AMHR2/tissue accessed on 18 December 2020). In order to confirm such an expression profile, we systematically investigated physiological AMHRII distribution in organs. Human AMHRII gene expression was measured by qRT-PCR in cDNA samples of 48 normal human tissues (Figure 1). Data presented as AMHRII/GAPDH ratios showed that AMHRII transcription was mainly restricted to the ovary but was also found in adrenal gland and testis. A minor level of transcription, six–eight-fold lower than that observed in ovary, was detected in penis and pancreas, whilst in the other organs tested, AMHRII transcription was either undetectable or more than 2000-fold lower than in ovary.

**Figure 2:**
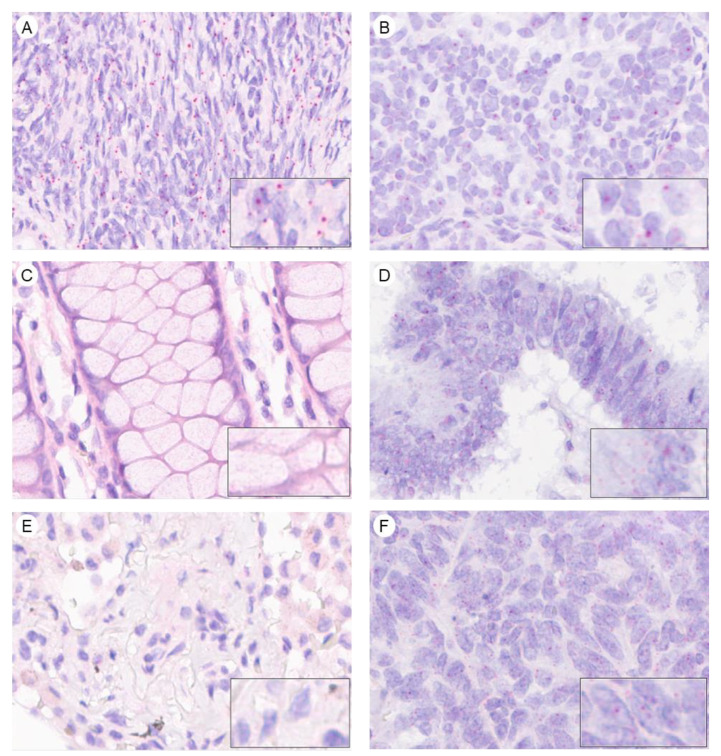
RNA in situ hybridization for semi-quantitative determination of mRNA expression levels of AMHRII in normal human tissues (**A**,**C**,**E**) and tumoral human tissues (**B**,**D**,**F**). Normal and tumoral tissues were stained with specific RNA AMHRII probes Hs-AMHRII (30ZZ). (**A**) Normal ovary tissue, RNAscope score 1, (**B**) Ovarian cancer, RNAscope score 1, (**C**) Normal colon, RNAscope score 0, (**D**) Colon cancer, RNAscope score 1, (**E**) Normal lung, RNAscope score 0, (**F**) Lung cancer, RNAscope score 1. Scoring was determined according to the manufacturer criteria: Score 0, no staining or less than 1 dot/10 cells; score 1, 1–3 dot/cell; score 2, 4–9 dots/cell; score 3, 10–15dots/cell; score 4 more than 15 dots/cell.

To complete this investigation with another technique, we designed a probe for detecting AMHRII transcription with the RNAscope assay, a recent in situ hybridization (ISH) technology. A Tumor Micro-Array (TMA) composed of 175 Formalin-fixed Paraffin Embedded (FFPE) samples from normal human organs was assessed and scores were determined by counting the number of colored dots per cell (Figure 2), according to the guidelines defined by the manufacturer. On this TMA, only 105 samples were analyzed by RNAscope due to missing or inadequate samples. These 105 samples were submitted to a Quality Control test using generic probes to eliminate samples with degraded RNA. As a result of this stringent procedure only 53 samples were finally evaluated for AMHRII expression. This experiment showed a lack of AMHRII transcription in most of the tissues tested (Supplementary Table S1): breast, exo-cervix, colon, endometrium, fallopian tube, gallbladder, ileum, renal medulla, liver, lung, lymph node, prostate, seminal vesicle, skin, spleen, muscular stomach, thyroid, tonsil, and uterus. A marginal staining, characterized by a score level of 1 in less than 20% of cells, was observed in a few samples of bladder, renal cortex, pancreas, placenta, stomach fundus, and thymus. A low level of transcription was also detected in pancreas with 20% of cells with score 1 in 2/2 samples tested. Conversely, robust AMHRII transcription, characterized by a score 1 in more than 50% of cells, was detected in tissues previously described as expressing the AMHRII gene, i.e., ovary, testis, and adrenal gland. Our results confirm and extend previous data generated by qRT-PCR and underline the fact that AMHRII expression is highly restricted in normal tissues.

### 2.2. AMHRII Transcription Is Detected in Gynecological and Non-Gynecological Cancer Tissues

Transcription of AMHRII in ovarian cancer was confirmed by ISH using the RNAscope assay as depicted in Figure 2. Thirteen ovarian cancer samples fixed on slides or TMA were tested and all of them were found to be positive for AMHRII transcription with scores of 1 or 2 in 40 to 80% of cells (data not shown). To investigate AMHRII expression in non-gynecological cancers, a TMA with a panel of cancers samples were tested by the RNAscope, in addition to individual slides of 4 major cancers (colon, kidney, liver, lung). This approach showed AMHRII mRNA transcription, characterized by a score of 1 or greater in 50% of cells, in several primary cancer tissues including hepatocarcinoma (5/9), melanoma (3/7), colorectal (11/17), lung (13/16), renal (6/13), and head and neck (3/5) cancer (Supplementary Table S2). Detection was marginal, with only one positive sample, in prostate, pancreas, bladder, and breast cancer. However, these preliminary studies were performed with a limited number of samples (from 4 to 16) and data would need to be extended to a larger panel of samples to determine more precisely a robust percentage of positive cases. Investigation regarding colorectal cancer was more specifically reinforced by a complementary study with 41 additional tumor samples. By cumulating these three studies (58 colorectal cancer samples), AMHRII mRNA was detected in 55% of cases.

### 2.3. AMHRII Protein Expression Is Detected in Gynecological and Non-Gynecological Cancer Tissues

To confirm AMHRII expression at the protein level, a procedure for detecting AMHRII in fixed tissue was established with biotinylated murlentamab. Those data are summarized in Table 1. Regarding gynecological cancers, a panel of 76 human ovarian tumors, 45 cervical cancers, and 185 endometrial cancer samples were analyzed. As previously observed by Bakkum-Gamez et al. (2008) [13], AMHRII was distributed at the cell membrane and into the cytoplasm. Due to this distribution, a Global Histological score (GHs) was established, considering both cytoplasmic (Cy) and membranous (Mb) AMHRII expression: GHs = (Mb Intensity × Mb Frequency) + (Cy Intensity × Cy Frequency). AMHRII was considered to be highly expressed when GHs > 1.5. A high expression of AMHRII was detected in 66% of ovarian cancer samples, and more specifically in the sub-group of 56 serous adenocarcinomas with 73% of cases highly expressing AMHRII. AMHRII expression in cervical and endometrial cancer panels was less marked with 42% and 18% of samples having high expression, respectively.

Concerning non-gynecological cancers, a panel of 631 samples corresponding to 15 different cancers was screened for AMHRII expression. As shown in the Table 1, AMHRII protein expression was observed in various solid tumors and especially in RCC, CRC, HCC, and NSCLC (examples of IHC staining are presented in the Supplementary Figure S1). The subtypes of each cancer were also presented but numbers of cases were too small to definitively conclude that there was a difference of frequency in comparison to the whole population. Overall, we did not find any correlation between AMHRII expression and tumor stage, grade, nor age of patients, although studies on larger panels will be needed to confirm this result. Interestingly, in terms of gender, AMHRII expression in NSCLC was stronger in women (67%) than in men (30%). However, this gender difference was not detected in other cancer types and should be confirmed using an extended cohort of lung cancer samples. In terms of cellular distribution, as observed by Beck et al. (2016) in NSCLC [23], AMHRII protein was detected at the membrane and into the cytoplasm with some exceptions, such as clear cell renal cancer where only membranous expression was detected. Further investigations are needed to understand the role of the intracellular pool of AMHRII.

IHC techniques were also used to detect AMHRII in a panel of 590 FFPE samples of PDX models coming from Xentech, Charles River, and CrownBio. Among the 28 different cancer types tested, a high level of expression was confirmed in ovarian cancer, RCC, and CRC (Supplementary Figure S2).

Pre-analytical conditions are important for the quality of AMHRII staining on FFPE samples. In the case of liver tumors, samples came from two different sources, US Biomax and Indivumed, and percentages of samples expressing AMHRII were significantly different, with 68% and 27% positive samples, respectively. Depending on the source, we also experimented with the appearance of a slight background staining. We hypothesized that AMHRII was sensitive to the fixative solution which was confirmed by treating spheroids of COV434-AMHRII cells for various incubation times with formalin (data not shown). This observation suggests that detection by IHC performed with routinely processed samples is likely to underestimate the level of AMHRII expression.

**Figure 3:**
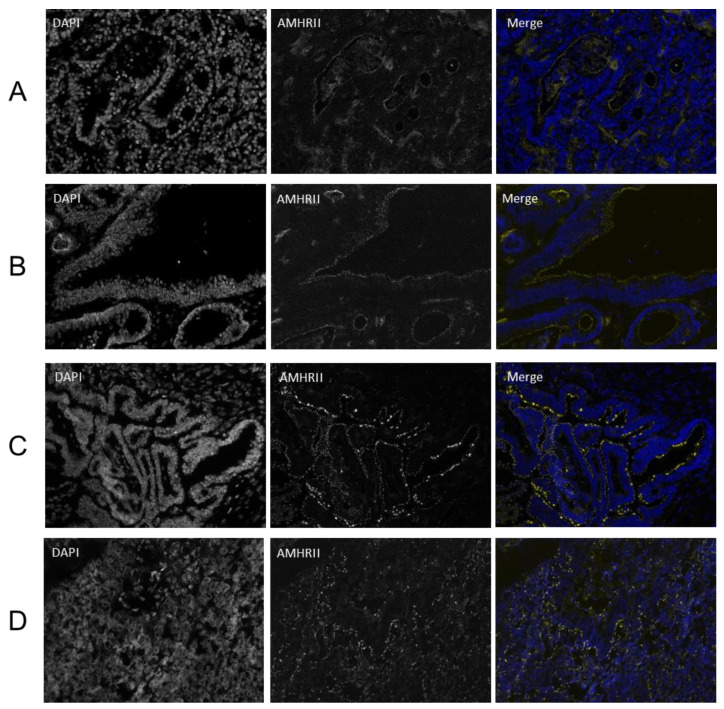
Immunofluorescence staining with murlentamab antibody of colorectal cancer ((**A**): A3764 Tp2, (**B**): A3742 Tc15, patient biopsies), ovarian cancer ((**C**): 1A717, (**D**): K583, patient biopsies). All fluorescent images were acquired with a widefield microscope DM5000 (Leica) with ×20 magnification. In Merge pictures, nuclear staining with DAPI was showed in blue and AMHRII staining with GM102 antibody was indicated in yellow.

To overcome the problem of reduced detection of AMHRII protein due to its hypersensitivity to formalin, AMHRII expression was confirmed in frozen and fresh samples. Indirect Immunofluorescence was performed on frozen tissue sections and we confirmed AMHRII expression in CRC tissue (Figure 3A,B) as well as in ovarian tumors (Figure 3C,D). A high density of strongly fluorescent dots was sometimes observed, the specific location (membrane or cytoplasm) of which was hard to assess because this staining was not amplificated as with IHC. Although some images suggested that the AMHRII receptor could form a cluster at the membrane, we cannot exclude from these immunofluorescence studies a potential intracellular AMHRII, as observed by IHC staining following a drastic antigen retrieval, a step which was not performed on frozen samples used for immunofluorescence detection.

**Figure 4:**
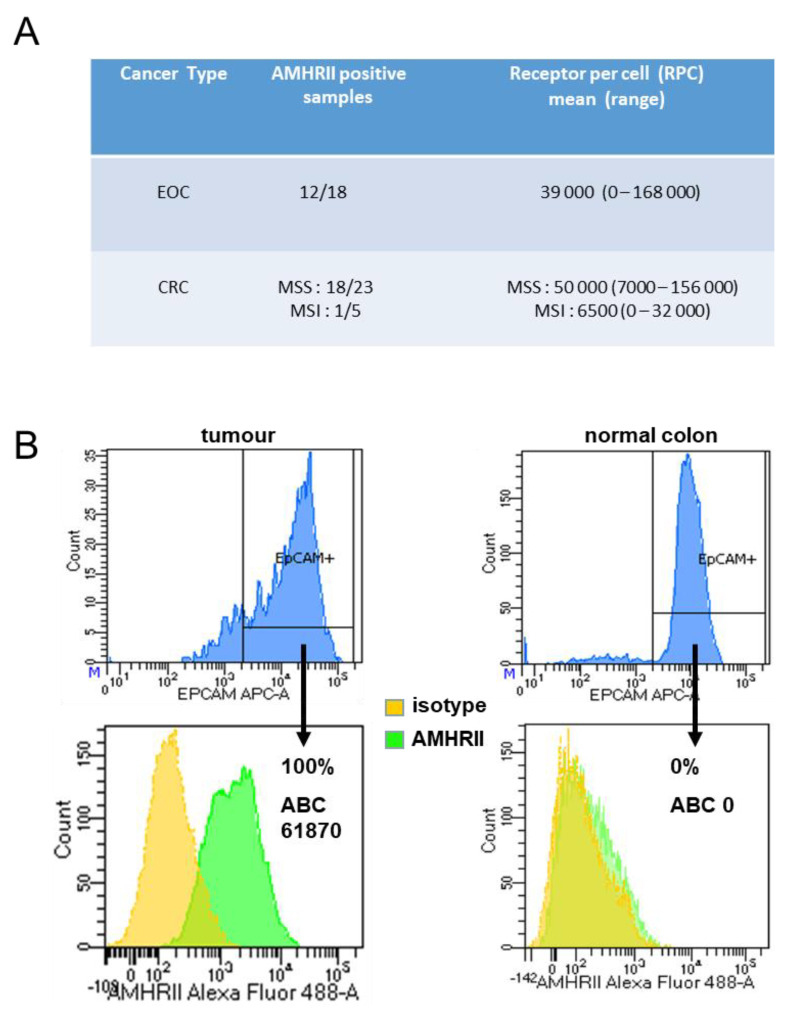
AMHRII expression by flow cytometry in ovarian and colorectal cancers. (**A**) The number of RPC was assessed as mentioned in the Material and Methods section. (**B**) representative images of AMHRII expression in EpCam+ cells in a MSS CRC and in its paired normal colonic mucosa.

In CRC, AMHRII expression was confirmed and quantified by flow cytometry using fresh samples of patient tumors. When we had enough materials we also confirmed this expression by Western-blotting (Figure S4). With this technique, we first controlled the specificity of murlentamab using COV434 and HC116 wild-type cell lines and cell lines transfected for overexpressing AMHRII (Figure S4A,B) then we confirmed in five CRC samples the positivity or negativity of AMHRII expression previously evaluated by flow cytometry (Figure S4C). The relatively unusual approach of flow cytometry was chosen due to the unstable nature of AMHRII expression on cell lines. AMHRII expression was assessed in the Epcam+ viable cells of CRC. Epcam (also named CD326) is commonly used in flow cytometry to assess epithelial cells, either normal or tumor epithelial cells. A diffuse, strong Epcam protein expression is typically observed in the vast majority of tumor cells from CRC samples. This selection permitted us to identify tumor cells from stroma cells and to compare AMHRII expression in both tumor and normal epithelial colon cells. In microsatellite stable (MSS) CRC, AMHRII was expressed in Epcam+ positive cells in 18/23 cases (78%) and was not expressed in five cases (22%). In the 18 AMHRII+ CRC, the mean of positive cells was 88% (range 32–100%). In addition, fluorescence quantification using calibration beads showed a significant number of receptors in Epcam+ tumor cells in all of the 18 MSS CRC (mean of receptors per cell (RPC) 50,000; range 7000–156 000) (Figure 4A,B). In comparison, the antibody binding capacity (ABC) values obtained with ovarian cancer samples were slightly lower (mean RPC 39,000; range 0–168 000) (Figure 4A). In contrast, AMHRII expression was either undetectable or low in most cases of the microsatellite unstable (MSI) CRC (4/5), both in terms of percentage of cells and RPC values (Figure 4A). However, this apparent difference in AMHRII expression profiles between MSS and MSI CRC samples would need to be confirmed in a larger cohort of CRC. Remarkably, in the paired normal colonic mucosa situated at a distance from the tumor, AMHRII was either not expressed or faintly expressed in the Epcam+ epithelial cells, both in terms of percent positive cells and RPC values (Figure 4B). Using the same tools, we performed flow cytometry analysis on a panel of samples of normal blood and hematological malignancies (five Acute Myeloid Leukemia, five Multiple Myeloma, and five Chronic Lymphoid Leukemia). None of these samples were positive for membranous AMHRII expression (data not shown).

### 2.4. AMHRII Protein as a Target for Antitumor Activity of Murlentamab, a Low Fucosylated Humanized Anti-AMHRII Antibody

**Figure 5:**
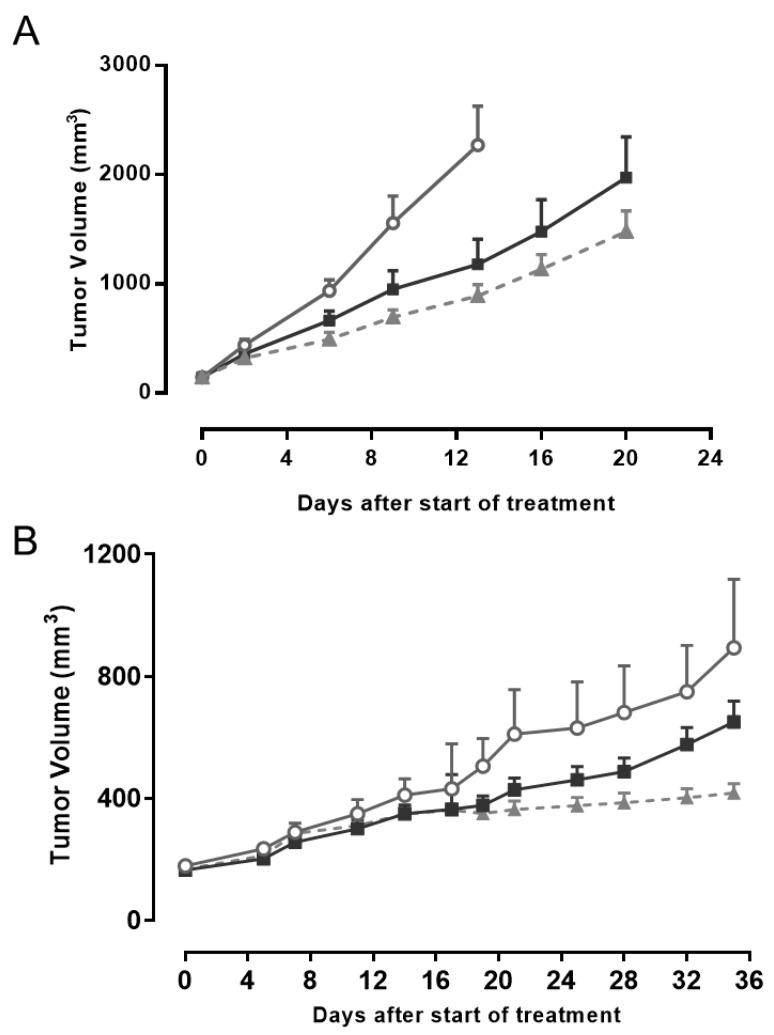
In vivo efficacy of murlentamab. (**A**) Tumor growth inhibition of murlentamab in the hepatocarcinoma PDX model (LI1097). Nude mice (n = 8/group) with established tumors (~145 mm^3^) were treated twice-weekly during 4 weeks by intravenous injection of murlentamab at 20 mg/kg. Sorafenib was administered p.o. at 50 mg/kg every day for 4 weeks. Open circle: vehicle, black square: murlentamab, Grey triangle: Sorafenib. (**B**) In vivo efficacy of murlentamab in the colorectal cancer PDX model (CTG-0401). Nude mice (n = 12/group) with established tumor (~175 mm^3^) were treated twice-weekly for four weeks by intravenous injection of murlentamab at 20 mg/kg. Irinotecan was injected i.p. at 100 mg/kg at day 1, 7 and 14. Open circle: vehicle, black square: murlentamab, Grey triangle: Irinotecan.

Murlentamab is a low fucosylated humanized anti-AMHRII antibody that demonstrated antitumor activity in vivo against ovarian cancer models by stimulating the immune system against tumors [21,22]. Murlentamab is currently under evaluation in a Phase 1 clinical trial in patients with AMHRII positive gynecological cancers (NCT02978755). Since AMHRII was also detected in PDX models of non-gynecological cancers (Supplementary Figure S2), we decided to perform an in vivo evaluation of murlentamab in these models. A difficulty of these preclinical studies lies in the stability of AMHRII expression since, as previously observed with ovarian cancer models [22], and for reasons not yet understood, a decrease of AMHRII expression is often observed after successive passages in vivo. A first study was conducted on the HCC PDX model LI1097 to evaluate the efficacy of murlentamab compared to that of sorafenib, standard of care for HCC used at its optimal scheme of dose and scheduling on this model, according to previous studies by CrownBio. As shown in Figure 5A, antitumor activity of murlentamab used at its optimal scheme of administration (based on previous studies [21,22]), was close to that of sorafenib, characterized by tumor growth inhibition (TGI) values of 54.3% and 64.9%, respectively. We observed a larger heterogeneity of response in the group treated with murlentamab, suggesting a heterogeneity of AMHRII expression from one grafted tumor sample to another. Importantly, murlentamab was clearly better tolerated than sorafenib in LI1097 tumor-bearing mice, since the mean maximum body weight losses were 0.38% and 7.63%, respectively. In a second study, we used the CRC xenograft PDX model CTG-0401 to assess the efficacy of murlentamab in comparison to irinotecan, a reference compound for CRC used at its optimal scheme of administration on this model, according to information from Champions. As shown in Figure 5B, antitumor activity of murlentamab was close to that of irinotecan, with TGI values on day 35 of 39% and 54%, respectively. As with the previous model, no significant body weight loss was noticed with murlentamab whilst there was a maximum body weight loss of 1.7% and one animal found dead at day 28 in the group treated with irinotecan. Overall, these in vivo experiments provided a preclinical rationale for moving into clinical trials with murlentamab in non-gynecological cancers.

## 3. Discussion

Few studies have carefully examined the expression of AMHRII in tumors. AMHRII protein and gene expression have been studied mainly in gynecologic cancers. At the Mayo Clinic, which led the first important IHC study on a large panel of EOC (182) and other gynecologic cancers, AMHRII was detected in nearly 70% of EOC, as well as in a majority of endometrial cancers (75%; 82/109) and ovarian dysgerminomas (77%; 17/22). This study used 12G4, a murine monoclonal antibody, from which murlentamab was derived and sharing the same target epitope [13]. More recently, an IHC study with a sheep polyclonal antibody applied to a tissue micro-array of 416 micro-dissected serous ovarian tumors found 86% of samples positive for AMHRII expression [17]. A limited number of other studies analyzing 20 to 40 samples each and using Western-blot, RT-PCR, or IHC with monoclonal, polyclonal antibodies, or biotinylated AMH confirmed AMHRII expression in 50% to 85% of EOC [16], 96% to 100% of GCT [24,25] [14,16], and 100% of cervical cancers [15]. All these studies undoubtedly demonstrated that AMHRII was expressed by gynecological cancers and our results, with slight differences depending on the technique used, confirm this observation.

AMHRII expression in tumors probably occurs during the de-differentiation process, characterized by re-expression of fetal proteins that is usually observed in carcinogenesis [26,27]. At this stage, one cannot exclude the activation by certain oncogenes such as WT1 which are highly expressed in ovarian cancer [28] and known to increase AMHRII expression by one of its isotypes designated “–KTS” [29].

Until now the expression of AMHRII in non-gynecological cancers has been scarcely reported in few cell lines derived from epithelial cancers such as breast cancer, prostate cancer, and ocular melanoma had not been robustly confirmed in clinical samples [30,31]. The first solid observation was presently in a study with few samples of NSCLC [23]. Our study provides the first demonstration of the expression of AMHRII in non-gynecological cancers, using a large panel of tumor samples and complementary methodological approaches. Indeed, AMHRII was found to be expressed at the plasma membrane in more than 50% of RCC, HCC, CRC, and NSCLC samples analyzed. Focusing our efforts on CRC, we confirmed AMHRII expression, both at the mRNA level by ISH in most cases (64.7%) and at the protein level using immunoperoxidase (53%) or immunofluorescence. Using flow cytometry on fresh tissues to quantify AMHRII expression at the plasma membrane, we showed that AMHRII is aberrantly expressed in CRC cells as compared to normal colonic epithelial cells in 70% of CRC with a mean receptor density of 50,000, a level even slightly higher than that found in ovarian cancers (39,000). This protein expression is relatively high in comparison to the level of mRNA transcription observed, indicating that post-translational regulation plays a major role in AMHRII expression [32]. Furthermore, our results suggest that AMHRII expression depends on an oncogenic pathway, since it is higher in MSS CRC samples than in MSI CRC samples. These data suggest that therapy with an anti-AMHRII antibody could constitute an interesting alternative in the subgroup of MSS CRC known to be refractory to innovative therapies such as immune checkpoint inhibitors. However, the lower expression profile observed in MSI CRC needs to be confirmed on a larger cohort.

We hypothesize that the broad expression of AMHRII expression in several cancer types might be related to the multifunctional endocrine role of the AMH/AMHRII pathway. Indeed, careful examination of embryological publications indicates that AMH most probably displays functions beyond its crucial role in gender determination. AMH was demonstrated to be a negative regulatory factor in fetal rat lung maturation [33] and has been suggested to display activity on tissues that share a common neural crest embryological origin [30]. Expression of AMHRII was also observed during differentiation of prostate and mammary cells [34,35]. In recent studies, a strong positive association has been observed between AMH and breast cancer risk, suggesting a role of AMH in breast cancer progression, especially in ER+ PR+ breast tumors [36,37]. Consequently, AMH and AMHRII could be re-expressed in many tumor types during their de-differentiation as suggested by gynecological cancers. Concerning CRC, no role of AMH/AMHRII in colon embryogenesis has been described so far. However, transcription of AMH and AMHRII in colon cancer were mentioned recently [38] and a negative impact of AMH on survival of patients with CRC has been suggested in a recent publication [39] and as well as on the Protein Atlas website (https://www.proteinatlas.org/ENSG00000104899-AMH/pathology accessed on 27 January 2021). We recently confirmed AMH protein expression by IHC in some CRC FFPE samples (Supplementary Figure S3). Interestingly, ovarian cancer stem cells have been shown to express AMHRII [40]. Therefore, the potential expression in cancer stem cells in other organs and a potential role in tumor relapse need to be addressed. Importantly, Beck et al., hypothesized a possible role of AMH/AMHRII in the epithelial-mesenchymal transition in lung cancers [23]. Further studies are needed to explore this hypothesis in CRC.

## 4. Materials and Methods

### 4.1. Cell Lines and Reagents

Cells from the human germ cell tumor cell line COV434 [41] were transfected with the cDNA encoding full-length human AMHRII in the pCMV6 plasmid to stably express AMHRII and to constitute the COV434-AMHRII cell line, as described by Kersual et al. (2014). Cells were grown in DMEM F12 medium containing 10% heat-inactivated fetal bovine serum, 0.1 mg/mL streptomycin, 0.1 IU/mL penicillin, and 0.25 µg/mL amphotericin B. COV434-AMHRII cells were supplemented with 0.33 mg/mL geneticin. Cells were grown at 37 °C in a humidified atmosphere with 5% CO_2_ and medium was replaced twice a week. All culture media and supplements were purchased from Thermo Fisher Scientific (Waltham, MA, USA—Gibco BRL).

### 4.2. Patient Specimens

All samples originated from patients that signed an informed consent according to the current regulations. For IHC study, FFPE tissue samples of renal, prostate, gastric, thyroid, and bladder cancer were provided by Indivumed. US Biomax provided samples of non-Hodgkin lymphomas and endometrium, lung (neuroendocrine), testis, and pancreas cancer. Samples of liver cancer were obtained from these two CROs and all other slides and TMAs were fixed in “AFA” solution (a mix of Alcohol, Formol and Acetic acid) at Institut Curie.

For flow cytometry analyses, 18 women with ovarian cancer undergoing surgery at Léon Bérard centre (Lyon) from July 2016 to April 2019 were prospectively included in this study. Fifteen blood samples from patients with hematological malignancies (five acute myeloid leukemia, five multiple myeloma and five chronic lymphoid leukemia) was also provided to be tested in parallel to blood sample of healthy donor from the Etablissement Français du Sang. Ovarian tumor cells were dissociated and analyzed by flow cytometry the same day. In addition, 28 CRC (23 MSS and 5 MSI) patients undergoing surgery at the Digestive Surgery Department of Nantes University Hospital from October 2017 to May 2019 were prospectively included in this study. The exclusion criteria were neoadjuvant treatment (radiotherapy and/or chemotherapy) and small tumor size (<2 cm). Clinicopathological data of CRC patients are summarized in Supplementary Table S3.

About 1cm^3^ of tumor and paired normal colonic mucosa (10 cm downstream the CRC tumor) were collected on the fresh surgical resection by the pathologist. The samples were either stored overnight at 4 °C in a Macs Tissue Storage solution from Miltenyi Biotec and processed for dissociation and flow cytometry analysis the next day or, for 10 cases, frozen in 10% DMSO in fetal bovine serum and then thawed and processed several days later.

### 4.3. Reverse Transcriptase Quantitative-PCR

Total RNA from 2–5 × 10^6^ cells pellet was prepared using Trizol^®^ Plus RNA Purification Kit (Ambion) according to the manufacturer’s instructions. RNA (1 µg) was reverse transcribed using Maxima H Minus First Strand cDNA Synthesis Kit (Thermoscientifics, K1651) and oligo-dT primers by incubation 10 min at 25 °C for priming and 15 min at 50 °C for transcription followed by 5 min at 85 °C for reverse transcriptase inactivation.

Quantitative PCR was performed in Light Cycler 480 (Roche) in 96-wells microplates using TaqMan gene Expression Master Mix (Applied Biosystem). To detect AMHRII, we used the human AMHRII Mix Hs010866508g1 (Applied Biosystem). The reference gene, human GAPDH, was detected with Mix 4310884e (Applied Biosystem). Amplifications were performed using cDNA template (100 ng equivalent RNA) and the following protocol: UDG pre-treatment 2 min at 50 °C, and denaturation 10 min at 95 °C followed by 40 cycles of 15 s at 95 °C/30 s at 60 °C/30 s at 70 °C. A melting curves analysis was performed at the end of each experiment to control for the absence of genomic DNA and dimer primers. Each cDNA samples and controls (“no template sample” and “no reverse transcript RNA”) were tested in duplicate. The mean values of Cycle Threshold (Ct) were calculated and the AMHRII relative quantification was expressed as 2^-∆∆Ct^ where ∆∆Ct = ∆Ct_sample_-∆Ct_calibrator_ and ∆Ct = Ct_AMHRII_-Ct_GADPH_.

### 4.4. In Situ Hybridization Using RNAscope

Before applying the RNAscope assay with target probes, each sample was quality controlled for RNA integrity with a probe specific to the housekeeping gene cyclophilin B, also named PeptidylProlyl Isomerase B. Only samples with an average of >4 dots per cell were included for analysis. Negative control background staining was evaluated using a probe specific to the bacterial dapB gene; only samples with an average of <1 dot per 10 cells were included for analysis.

Paired double-Z oligonucleotide probes were designed against target RNA using custom software as described previously [42]. GenBank accession numbers, number of probe pairs and probe regions were: NM_020547.3, 30 probe pairs, 122–1512 nt.

Detection of RNA transcripts was performed with the RNAscope^®^ 2.5 HD Red Reagent kit (Advanced Cell Diagnostics) according to the manufacturer’s instructions. RNA staining signal was identified as red and green punctate dots. Bright field images were acquired using a Zeiss Axio Imager M1 microscope using a 40× objective. Scoring was determined according to the manufacturer criteria: Score 0, no staining or less than 1 dot/10 cells; score 1, 1–3 dots/cell; score 2, 4–9 dots/cell; score 3, 10–15 dots/cell; score 4 more than 15 dots/cell.

### 4.5. Immunohistochemistry

Routinely Formalin-fixed, paraffin-embedded, 3 μm-thick tissue sections were de-paraffinized, rehydrated, and then unmasked in target retrieval solution at pH9. Anti AMHRII biotinylated detection was performed by immune-peroxidase technique and 3,3′-diaminobenzidine tetrahydrochloride hydrate (DAB) chromogenic substrate revelation. After blocking endogenous peroxidase activity and inhibiting non-specific staining, the slides were incubated with diluted antibody (1/200) for 60 min at room temperature. The tissue sections were then washed with phosphate buffered saline solution (TBS) and incubated with a vectastain elite ABC kit for 8 min. Immunoreactive signals were detected using DAB substrate solution. Finally, the sections were lightly counterstained with Mayer’s Hematoxylin. Images acquisition was performed using IMS software (Philips Digital Pathology).

Cellular expression of AMHRII was performed on 18 different types of malignant tumors. As COV434-AMHRII cells, used as positive control, most of the cells expressing AMHRII showed diffuse, predominantly cytoplasmic immunostaining, with a moderate to strong intensity and constant membranous staining. This was in accordance with previous observations suggesting a high pool of intracellular AMHRII receptors in cancer cells [43] and regulation of AMHRII expression by intracellular retention [32]. Due to this distribution, a GHs was established, considering both cytoplasmic (Cy) and membranous (Mb) AMHRII expression: GHs = (Mb Intensity × Mb Frequency) + (Cy Intensity × Cy Frequency). Examples of staining with GHs value from 0 to 3 are shown in Supplementary Figure S5.

### 4.6. Immunofluorescence

Frozen section of 7 µm were fixed with acetone for 10 min at −20 °C. The fixed sections were incubated with blocking buffer containing 10% goat serum/2% BSA/0.5% Triton ×100 for 30 min at room temperature. After the blocking step, slides sections were incubated with 3C23K-AF488 at 10 µg/mL conjugated antibody, then with anti-AF488 rabbit antibody (1:500, Invitrogen) followed by goat anti-rabbit AF647 (1:1000, Invitrogen) to obtain a well-defined AMHRII labelling and a reduced background staining. All antibodies were diluted in blocking buffer and incubated for 30 min at room temperature. Slides sections were washed three times with PBS-Triton 0.05% after each incubation. For negative controls, an isotype control R565-AF488 was used. The nuclei were stained with DAPI (Sigma Aldrich). The slides were mounted under coverslips (Knittel Glass) with DAKO Fluorescent mounting medium. Images acquisition was performed using fluorescence microscope Leica DM5000B equipped with the CoolSnap EZ CCD camera controlled by the Metavue software (Molecular Devices). Post-treatment images were performed using the ImageJ software (NIH; Bethesda, MD, USA).

### 4.7. Flow Cytometry

Fragments of tumor and paired normal colonic mucosa from CRC were cut into small pieces (1–2 mm^3^) in RPMI 1640 medium and underwent non-enzymatic mechanical dissociation using a glass Dounce tissue grinder or, for CRC tissues, the Miltenyi Gentle Macs dissociator. The homogenates were then filtered onto a 40 µm cell strainer, centrifuged and cells were counted on a hemocytometer.

For staining, approximately 100,000 ovarian cells or 300,000 colon cells were incubated for 30 min at 4 °C in a mixture of the following antibodies: 3C23K-AF488 (from GamaMabs) or isotype control (R565-AF488 from GamaMabs) together with a viability marker (FVS from Becton Dickinson) and a marker of epithelial cells for colon cells (Epcam-APC from Miltenyi reference 130-098-118).

In each experiment, the ovarian cell line COV434 transfected with AMHRII was used as a positive control. Colon and ovary cells (300,000 and 500,000, respectively) were incubated with either 3C23K-AF488 or the isotype control R565-AF488. After washing in PBS/0.1% BSA, stained cells were acquired in the viable cell gate on a flow cytometer using BD Diva software and further analyzed with Diva software. Cells and calibration beads were analyzed using the same instrument settings.

Quantification of AMHRII cell surface expression in cells from the tumors or paired normal colon as well as in COV434-AMHRII cells was performed using the Quantum™ Simply Cellular^®^kit (from Bang Labs reference 816-5), according to the manufacturer’s instructions. The calibrated beads were incubated with 3C23K-AF488 at a saturating concentration determined in preliminary experiments as 50 µg/mL or 10 µg/mL, for 30 min at room temperature or at 4 °C for colon and ovary cells, respectively. A calibration curve was generated by plotting the median of fluorescence of each bead population *versus* its assigned ABC, using the QuickCal^®^ software. ABC values, representing the RPC, were assigned to stained cell samples using the standard curve generated with the beads. Only Epcam-positive CRC cells were considered for analysis in order to exclude non-epithelial cells. The background signal represented by the isotype control was subtracted from the signal measured for 3C23K-AF488 in the analysis.

### 4.8. Western Blot

For western blotting, lysate cell extracts from HCT116 wild-type without any expression of AMHRII, COV434 wild-type with marginal expression of AMHRII and from HCT116 and COV434 clones transfected for expressing AMHRII were prepared with RIPA buffer (25 mM Tris-HCl pH 7.6, 150 mM NaCl, 1% NP-40, 1% sodium deoxycholate, 0.1% SDS) supplemented with EDTA 0.5 mM and Halt Cocktail Protease Inhibitor (Pierce). For CRC tissue samples a Precellys Tissue homogenizer was used after RIPA extraction. Protein quantification was done using Biorad Protein Assay Dye reagent concentrate (Biorad) with BSA calibration standard curve.

Samples were heated 10 min at 70 °C in presence of Nu-PAGE LDS Buffer with or without reducing agent (Thermo Scientific). Protein extracts (10 µg for cell extracts and 15 µg for tissue sample) were electrophoretically separated on NuPAGE Novex Bis-Tris Gels 12% in reduced conditions then transferred onto polyvinyl difluoride membrane (PVDF). Membranes were saturated overnight at 4 °C with PBS-Milk 5%. Membranes were incubated for 1h30 with 3C23K antibody diluted in PBS-Milk 5%-Tween 0.01%. The anti-AMHRII 3C23K antibody was detected with HRP conjugated goat F(ab’)2 anti-Human IgG F(ab’)2 (Jackson Immunoresearch, 109-036-006). Bands were detected using Super Signal West Dura Extended Duration Substrate (Thermo Scientific).

### 4.9. In Vivo Experiments

All in vivo studies were carried out in compliance with American Association for Assessment and Accreditation of Laboratory Care guidelines and with Institutional Animal Care and Use Committee of CrownBio or of Champions Oncology for study with, respectively, LI1097 or CTG-0401 models. Mice were housed 4–5 mice/cage on 100% virgin kraft nesting enrichment sheets in HEPA ventilated cages on a 12-12-h light-dark cycle at 20–23 °C and 30–70% humidity. Animals had access to water and an irradiated test rodent diet *ad libitum*.

PDX models were chosen after confirmation of AMHRII expression by at least two techniques. Both models were positive for AMHRII transcription determined by RNA sequencing with amplification factors of 2.14 and 33.38 for LI1097 HCC model and CTG-0401 CRC model, respectively. AMHRII protein expression was confirmed on LI1097 PDX section by IHC, and on CTG-0401 PDX section by immunofluorescence (Supplementary Figure S6).

Murlentamab was diluted in PBS and administered intraperitoneally, twice a week, for 4 weeks. The control groups in both experiments were treated with PBS (vehicle) with the same schedule of administration as murlentamab. Sorafenib (MelonePharma) was administrated *per os* at a dose of 50 mg/kg in water with 5% dextrose every day for 4 weeks. Irinotecan (Teva Parenteral Medicine Inc.) was administered intraperitoneally at a dose of 100 mg/kg at days 1, 7, and 14. In the study with LI1097 model, 7–8 week-old female BALB/c nude mice (HFK) were used whilst 5–8 week-old male Athymic Nude-*Foxn1^nu^* (Envigo) mice were engrafted with CTG-0401 tumor fragments. When tumors grafted in the flank (after anesthesia with isoflurane) reached a size about 150 mm^3^, mice were randomly assigned to control or treatments groups with 8–10 mice per group. Tumor growth was evaluated by measuring with a caliper two perpendicular tumor diameters and TGI were calculated according to standard method [44].

For both studies, in vivo toxicity was assessed by weight measurement. A treatment was considered as toxic and consequently stopped if a bodyweight loss of 15% or more persisted for three consecutive days, or if body weight loss reached 20%. Animals were sacrificed by CO_2_ inhalation followed by cervical dislocation at the end of the study or if the treatment was found to be toxic.

## 5. Conclusions

Overall, our data pave the way for new therapeutic approaches targeting AMHRII, in non-gynecological cancers. In vivo studies with murlentamab confirmed the potential antitumor activity of such an agent by showing tumor growth delay in PDX models of HCC and CRC. Better efficacy could be expected in humans since the antitumor activity of this antibody depends on the activation of the immune system [21,22] whilst these studies were performed in immune-deficient mice. Studies on humanized animals with functional human macrophages will be a way to better evaluate the antitumor potential of murlentamab. Globally these preclinical data obtained in parallel to the ongoing Phase 1 trial of murlentamab in gynecological cancers (NCT02978755) showing hints of activity as well as an excellent tolerability, have been the basis to initiate an exploratory Phase IIa study in metastatic CRC patients (NCT03799731), opening new perspectives of therapeutic application for this anticancer agent.

## 6. Patents

Patents WO/2018/189379 and WO2018/189381 resulted from the work reported in this manuscript.

## Figures and Tables

**Table 1:** Detection of AMHRII expression by IHC.

Cancer Type	Subtype	Number of Samples	% of Samples with GHs ≥ 1.5
**Adrenocortical cancer**		**4**	**75**
**Renal cell carcinoma**		**34**	**71**
	Clear cell carcino.	33	73
	Papillary carcino.	1	0
**Ovarian cancer**		**76**	**66**
	Serous adeno.	56	73
	Mucinous adeno.	6	50
	Endometrioid adeno.	5	40
	Clear cell adeno.	5	80
	Carcinosarcoma	2	50
	Metastatic adeno.	1	0
	Undifferentiated	1	0
**Colorectal cancer**		**30**	**53**
	Adeno.	27	56
	Mucinous adeno.	2	50
	Carcino.	1	0
**Liver cancer**	Hepatocarcinoma	**62**	**53**
**Non-small cell lung cancer**		**18**	**44**
	Large cell carcino.	4	50
	Sarcomatoid carcino.	1	100
	Adeno.	9	56
	Squamous cell carcino.	4	0
**Cervical cancer**		**45**	**42**
	Adeno.	5	0
	Adenosquam. carcino.	2	100
	Squamous cell carcino.	35	43
	Undifferentiated carcino.	3	66
**Testicular cancer**		**48**	**27**
	Spermatocytoma	42	31
	Spermatocytic seminoma	3	33
	Anaplastic spermacytoma	3	0
**Breast cancer**		**102**	**26**
	Inv. ductal carcino. Her2+	38	32
	Inv. ductal carcino. lum. A	9	11
	Inv. ductal carcino. lum. B Her2−	3	0
	Inv. ductal carcino. lum. B Her2+	5	0
	Inv. ductal carcino. lum. TN	42	26
	Medullary carcino. TN	5	60
**Thyroid cancer**		**19**	**21**
	Papillary	11	9
	Follicular	2	50
	Medullary	2	0
	Anaplastic	3	33
	Not specified	1	0
**Gastric cancer**	Adeno.	**40**	**20**
**Endometrial cancer**		**185**	**18**
	Endometrioid adeno.	130	22
	Adenosquam. carcino.	9	1
	Chorionic carcino.	4	0
	Clear cell carcino.	2	0
	Polypoidal endometrial carcino.	1	0
	Squam. cell carcino.	4	0
	Stromal sarcoma	6	0
	Non specified	29	14
**Bladder**		**19**	**11**
	Transitional cell carcino.	15	13
	Squam. cell carcino.	2	0
	Neuroendo. Small cell carcino.	2	0
**Pancreatic cancer**		**78**	**9**
	Ductal carcino.	69	9
	Adenosquam. carcino.	9	11
**Prostate cancer**		**30**	**7**
	Acinar adeno.	4	0
	Other adeno.	26	8
**Head & Neck cancer**	Epidermoid carcino.	**52**	**2**
**Small cell lung cancer**	Neuroendo. carcino.	**76**	**1**
**Non-Hodgkin’s lymphoma**		**18**	**0**

adeno., adenocarcinoma; carcino., carcinoma; lum., luminal; neuroendo., neuroendocrine; TN, triple negative; squam., squamous.

## Data Availability

All data generated in this study are shown in this article, its tables, and figures. Original or digitalized stained slides and TMAs are stocked at Institut Curie or at GamaMabs Pharma and are available on request from the corresponding author.

## Supplementary Materials

The following are available online at https://www.mdpi.com/article/10.3390/biology10040305/s1, Figure S1: AMHRII immunostaining in a panel of malignant tumors (×400). (A) and (B): granulosa ovary tumor, (C): breast HER2 carcinoma, (D): breast triple negative carcinoma, (E): colon carcinoma, (F): cortico-surrenaloma, (G): prostate carcinoma, (H): endometrial carcinoma, (I): cervical carcinoma, (J): pancreatic carcinoma, (K): liver carcinoma, (L): head and neck carcinoma, (M): lung carcinoma, (N): urothelial carcinoma, (O): kidney carcinoma, Figure S2: AMHRII immunostaining in a panel of PDX models evaluated by GHs scoring. For each pathology, number of PDX tested, mean value and range of AMHRII global score are specified, Figure S3: Examples of AMH detection in FFPE tumor samples of 3 patients with CRC characterized by (A) 25% of cells at 2+, (B) 40% of cells at 2+ and (C) 15% of cells at 2+. AMH detection was performed on a Leica Bond III with 5/6 clone anti-AMH monoclonal antibody from Genetex, diluted at d20 and detected by DAB chromogen. Right pictures are 40-fold magnifications of boxes indicated on the left, Figure S4: Western Blots to detect AMHRII protein with 3C23K/Murlentamab antibody. A) AMHRII detection using total extracts of COV434 wild-type (Wt) and COV434 overexpressing AMHR2 (R2+). B) AMHRII detection using total extracts of HCT116 wild-type (Wt) and of HCT116 clones (cl6 and cl22) transfected for overexpressing 2 different levels of AMHRII. C) AMHRII detection using total extract of CRC samples from 5 patients (P1, P5, P8b, P14 and P4). Figure S5: Examples of scoring by GHs for evaluating AMHRII expression in tumors: (A) score 0: absence of staining; (B) score 1: low staining; (C) Score 2: moderate staining; (D) Score 3: strong staining, Figure S6: (A) Immunohistochemistry staining of AMHRII with GM102 antibody of hepatocellular carcinoma PDX LI097. (B) Immunofluorescence staining with GM102 antibody PDX colorectal cancer CTG-0401. All fluorescent images were acquired with a widefield microscope DM5000 (Leica) with ×20 magnification. In Merge pictures, nuclear staining with DAPI was showed in blue and AMHRII staining with GM102 antibody was indicated in yellow, Table S1: Detection of AMHRII transcription by RNAscope in a FFPE TMA of human normal tissues, Table S2: Detection of AMHRII transcription by RNAscope in a FFPE multi-tumor TMA and additive individual slides, Table S3. Clinicopathological features of CRC patients.
