# Supplementary material for: The Expression of Anti-Müllerian Hormone Type II Receptor (AMHRII) in Non-Gynecological Solid Tumors Offers Potential for Broad Therapeutic Intervention in Cancer
Source: Biology (Basel). 2021 Apr 7;10(4):305. doi: 10.3390/biology10040305 (PMC8067808; doi:10.3390/biology10040305)
Supplement: Supplementary file 1 [file biology-10-00305-s001.zip › biology-1127192- Sup Fig 1.pptx]

## Slide 1
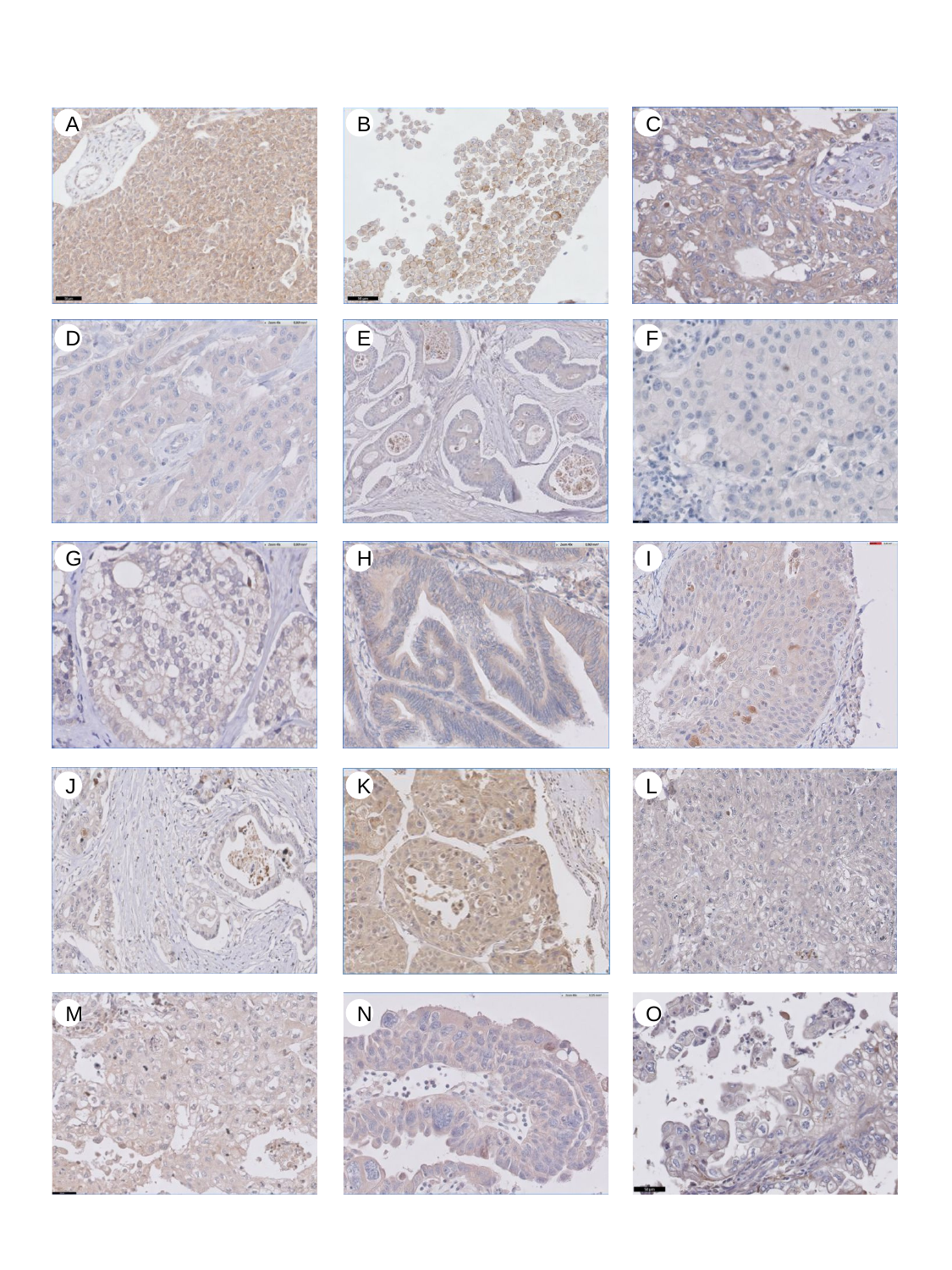

A
B
C
D
E
F
G
H
I
J
K
L
M
N
O

## Slide 2
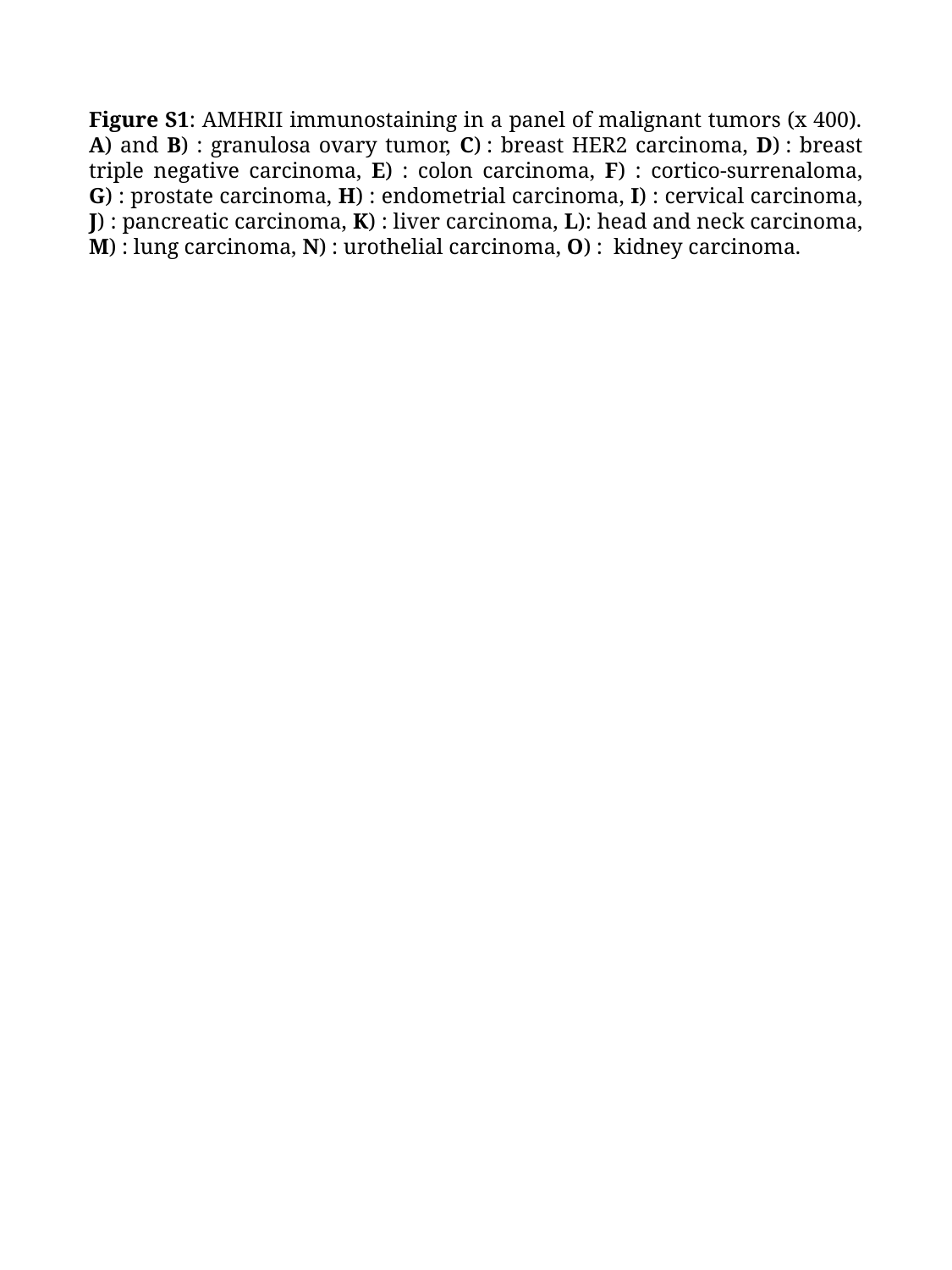

Figure S1: AMHRII immunostaining in a panel of malignant tumors (x 400). A) and B) : granulosa ovary tumor, C) : breast HER2 carcinoma, D) : breast triple negative carcinoma, E) : colon carcinoma, F) : cortico-surrenaloma, G) : prostate carcinoma, H) : endometrial carcinoma, I) : cervical carcinoma, J) : pancreatic carcinoma, K) : liver carcinoma, L): head and neck carcinoma, M) : lung carcinoma, N) : urothelial carcinoma, O) : kidney carcinoma.
