# Supplementary material for: The Expression of Anti-Müllerian Hormone Type II Receptor (AMHRII) in Non-Gynecological Solid Tumors Offers Potential for Broad Therapeutic Intervention in Cancer
Source: Biology (Basel). 2021 Apr 7;10(4):305. doi: 10.3390/biology10040305 (PMC8067808; doi:10.3390/biology10040305)
Supplement: Supplementary file 1 [file biology-10-00305-s001.zip › biology-1127192- Sup Fig 2.pptx]

## Slide 1
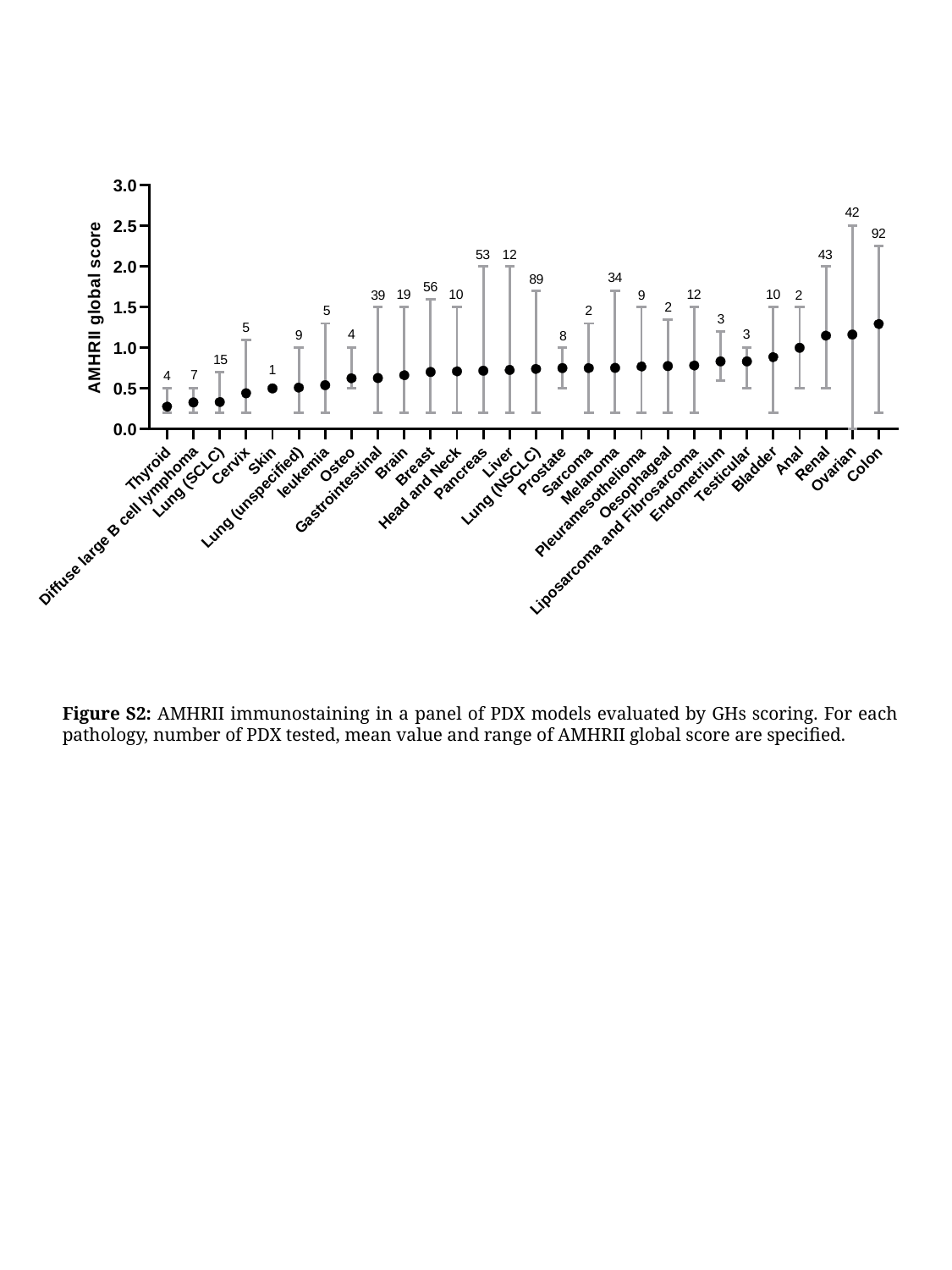

Figure S2: AMHRII immunostaining in a panel of PDX models evaluated by GHs scoring. For each pathology, number of PDX tested, mean value and range of AMHRII global score are specified.
