# Supplementary material for: The Expression of Anti-Müllerian Hormone Type II Receptor (AMHRII) in Non-Gynecological Solid Tumors Offers Potential for Broad Therapeutic Intervention in Cancer
Source: Biology (Basel). 2021 Apr 7;10(4):305. doi: 10.3390/biology10040305 (PMC8067808; doi:10.3390/biology10040305)
Supplement: Supplementary file 1 [file biology-10-00305-s001.zip › biology-1127192- Sup Fig 3.pptx]

## Slide 1
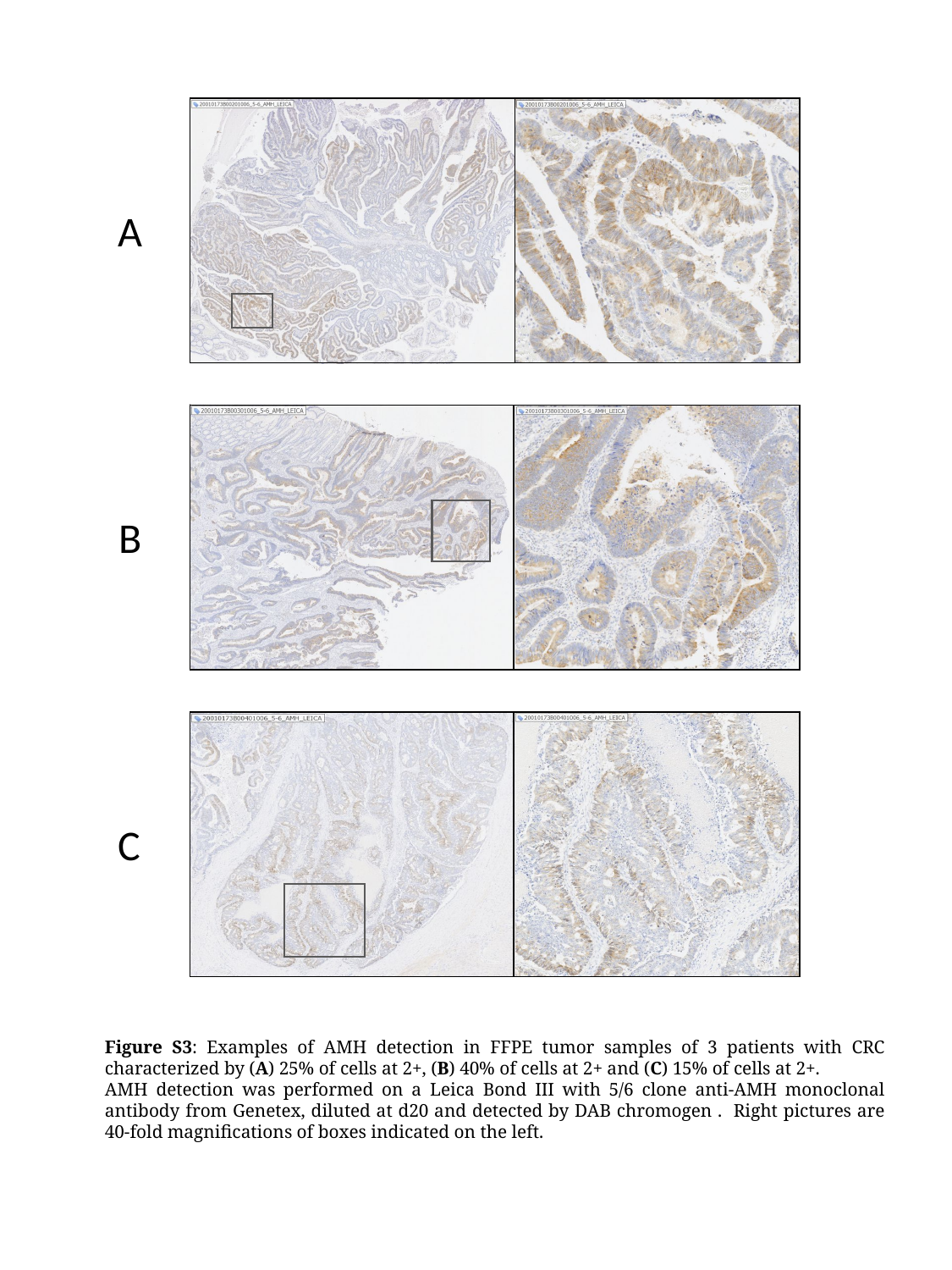

A
B
C
Figure S3: Examples of AMH detection in FFPE tumor samples of 3 patients with CRC characterized by (A) 25% of cells at 2+, (B) 40% of cells at 2+ and (C) 15% of cells at 2+.
AMH detection was performed on a Leica Bond III with 5/6 clone anti-AMH monoclonal antibody from Genetex, diluted at d20 and detected by DAB chromogen . Right pictures are 40-fold magnifications of boxes indicated on the left.
