# Supplementary material for: The Expression of Anti-Müllerian Hormone Type II Receptor (AMHRII) in Non-Gynecological Solid Tumors Offers Potential for Broad Therapeutic Intervention in Cancer
Source: Biology (Basel). 2021 Apr 7;10(4):305. doi: 10.3390/biology10040305 (PMC8067808; doi:10.3390/biology10040305)
Supplement: Supplementary file 1 [file biology-10-00305-s001.zip › biology-1127192- Sup Fig 4_New.pptx]

## Slide 1
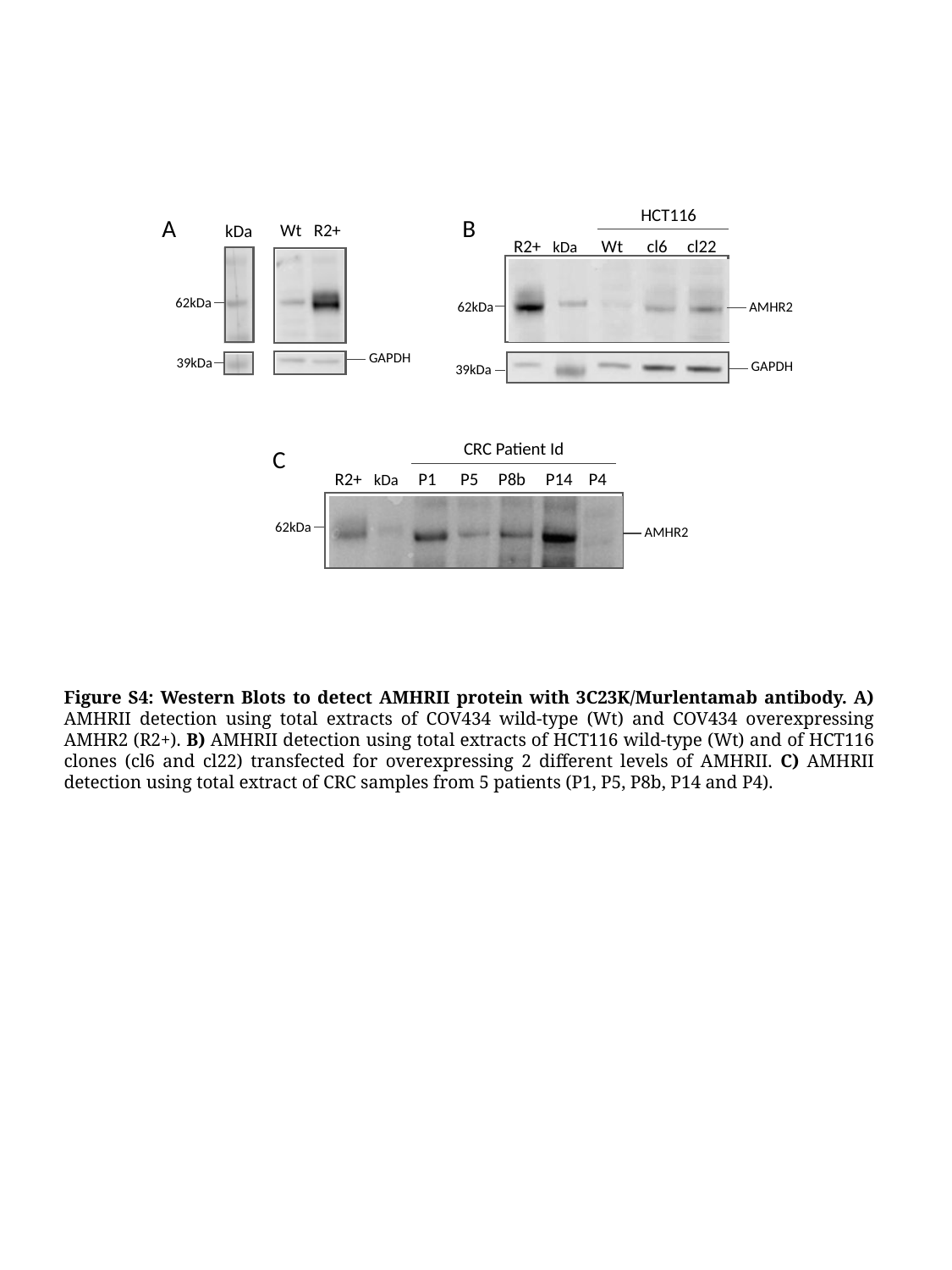

HCT116
B
R2+ kDa Wt cl6 cl22
AMHR2
62kDa
GAPDH
39kDa
A
 Wt R2+
kDa
62kDa
39kDa
GAPDH
CRC Patient Id
C
R2+ kDa P1 P5 P8b P14 P4
62kDa
AMHR2
Figure S4: Western Blots to detect AMHRII protein with 3C23K/Murlentamab antibody. A) AMHRII detection using total extracts of COV434 wild-type (Wt) and COV434 overexpressing AMHR2 (R2+). B) AMHRII detection using total extracts of HCT116 wild-type (Wt) and of HCT116 clones (cl6 and cl22) transfected for overexpressing 2 different levels of AMHRII. C) AMHRII detection using total extract of CRC samples from 5 patients (P1, P5, P8b, P14 and P4).
