# Supplementary material for: The Expression of Anti-Müllerian Hormone Type II Receptor (AMHRII) in Non-Gynecological Solid Tumors Offers Potential for Broad Therapeutic Intervention in Cancer
Source: Biology (Basel). 2021 Apr 7;10(4):305. doi: 10.3390/biology10040305 (PMC8067808; doi:10.3390/biology10040305)
Supplement: Supplementary file 1 [file biology-10-00305-s001.zip › biology-1127192- Sup Fig 5_New.pptx]

## Slide 1
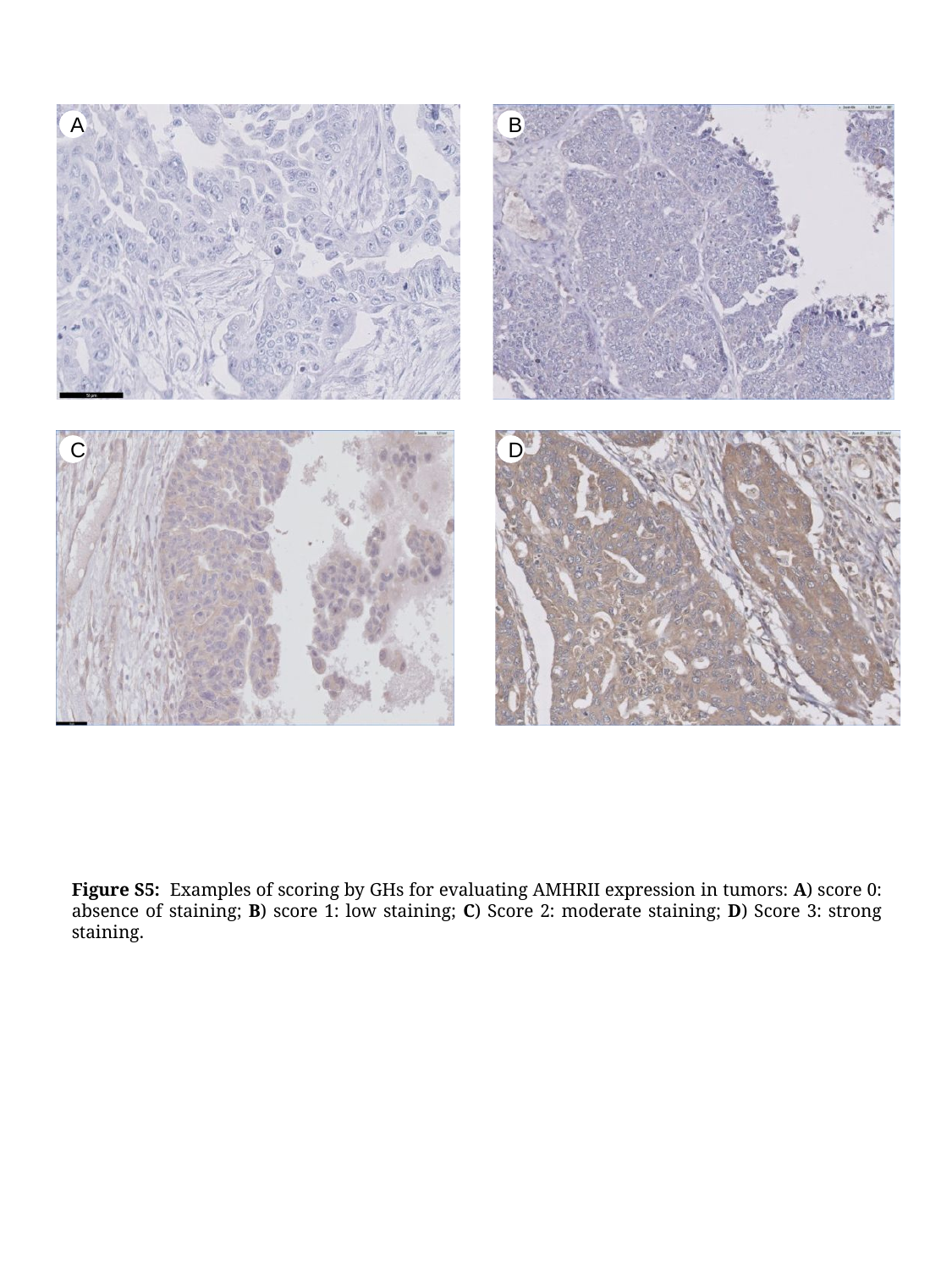

B
A
D
C
Figure S5: Examples of scoring by GHs for evaluating AMHRII expression in tumors: A) score 0: absence of staining; B) score 1: low staining; C) Score 2: moderate staining; D) Score 3: strong staining.
