# Supplementary material for: The Expression of Anti-Müllerian Hormone Type II Receptor (AMHRII) in Non-Gynecological Solid Tumors Offers Potential for Broad Therapeutic Intervention in Cancer
Source: Biology (Basel). 2021 Apr 7;10(4):305. doi: 10.3390/biology10040305 (PMC8067808; doi:10.3390/biology10040305)
Supplement: Supplementary file 1 [file biology-10-00305-s001.zip › biology-1127192- Sup Fig 6_New.pptx]

## Slide 1
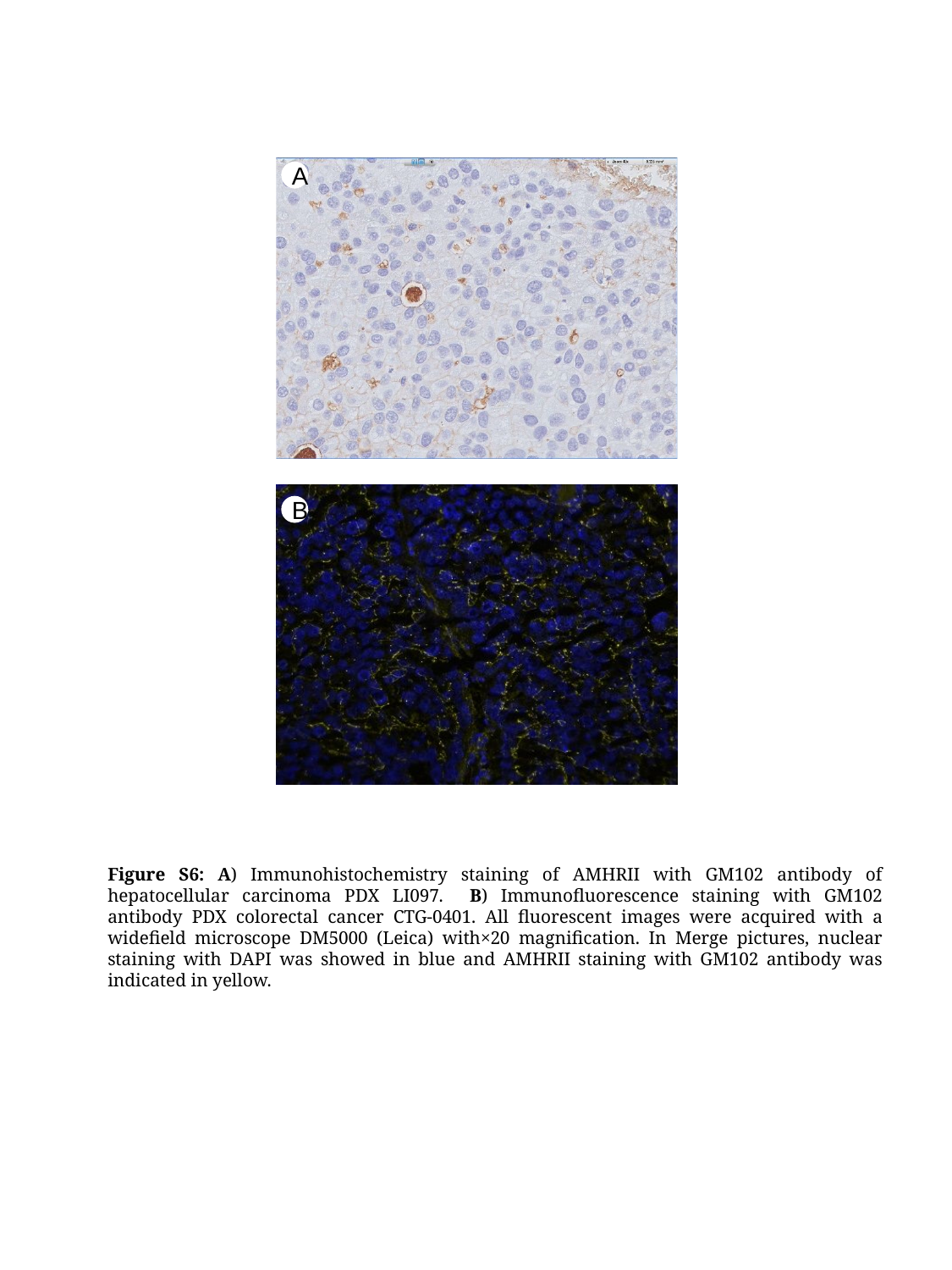

A
B
Figure S6: A) Immunohistochemistry staining of AMHRII with GM102 antibody of hepatocellular carcinoma PDX LI097. B) Immunofluorescence staining with GM102 antibody PDX colorectal cancer CTG-0401. All fluorescent images were acquired with a widefield microscope DM5000 (Leica) with×20 magnification. In Merge pictures, nuclear staining with DAPI was showed in blue and AMHRII staining with GM102 antibody was indicated in yellow.
