# Supplementary material for: The Expression of Anti-Müllerian Hormone Type II Receptor (AMHRII) in Non-Gynecological Solid Tumors Offers Potential for Broad Therapeutic Intervention in Cancer
Source: Biology (Basel). 2021 Apr 7;10(4):305. doi: 10.3390/biology10040305 (PMC8067808; doi:10.3390/biology10040305)
Supplement: Supplementary file 1 [file biology-10-00305-s001.zip › biology-1127192- Sup Table 1.docx]

**Table S1:** Detection of AMHRII transcription by RNAscope in a FFPE TMA of human normal tissues

| Tissue Type | Number of  evaluable samples | Number of  positive samples  with Score ≥ 1 | Number of samples  with ≥ 20% positive cells |
| --- | --- | --- | --- |
| Adrenal Gland | 3/3 | 2 | 1 |
| Bladder | 2/3 | 1 | 1 |
| Breast | 1/3 | 0 | 0 |
| Cerebellum | 0/3 | - | - |
| Cerebrum | 0/3 | - | - |
| Cervix (endo-) | 0/3 | - | - |
| Cervix (exo-) | 1/3 | 0 | 0 |
| Colon | 1/3 | 0 | 0 |
| Endometrium | 2/3 | 0 | 0 |
| Fallopian tube | 1/3 | 0 | 0 |
| gallbladder | 1/3 | 0 | 0 |
| heart | 0/3 | - | - |
| Ileum | 2/3 | 0 | 0 |
| Kidney (medulla) | 1/3 | 0 | 0 |
| Kidney (cortex) | 3/3 | 1 | 0 |
| Liver | 2/3 | 0 | 0 |
| Lung | 2/3 | 0 | 0 |
| Lymph node | 2/3 | 0 | 0 |
| Ovary | 1/3 | 1 | **1** |
| Pancreas | 2/3 | 2 | 0 |
| Parotid gland | 0/3 | - | - |
| Placenta | 3/3 | 1 | 0 |
| Prostate | 2/3 | 0 | 0 |
| Seminal vesicle | 3/3 | 0 | 0 |
| Skeletal muscle | 0/3 | - | - |
| Skin | 1/3 | 0 | 0 |
| Spleen | 1/3 | 0 | 0 |
| Stomach (fundus) | 2/3 | 1 | 0 |
| Stomach (muscular) | 1/3 | 0 | 0 |
| Testis | 3/3 | 3 | **3** |
| Thymus | 3/3 | 1 | 0 |
| Thyroid | 1/3 | 0 | 0 |
| Tonsil | 3/3 | 0 | 0 |
| Ureter | 0/3 | - | - |
| Uterus | 2/3 | 0 | 0 |
