# Supplementary material for: The Expression of Anti-Müllerian Hormone Type II Receptor (AMHRII) in Non-Gynecological Solid Tumors Offers Potential for Broad Therapeutic Intervention in Cancer
Source: Biology (Basel). 2021 Apr 7;10(4):305. doi: 10.3390/biology10040305 (PMC8067808; doi:10.3390/biology10040305)
Supplement: Supplementary file 1 [file biology-10-00305-s001.zip › biology-1127192- Sup Table 2.docx]

**Table S2**: Detection of AMHRII transcription by RNAscope in a FFPE multi-tumor TMA and additive individual slides

| Cancer Type | Number of  evaluable samples | Number of  positive samples  with Score ≥ 1 | Number of samples  with ≥ 50% positive cells |
| --- | --- | --- | --- |
| Bladder | 4 | 2 | 0 |
| Breast | 9 | 2 | 1 |
| Colorectal | 17 | 15 | 11 |
| Head & Neck | 5 | 4 | 3 |
| Kidney | 13 | 7 | 6 |
| Liver | 9 | 8 | 5 |
| Lung | 16 | 15 | 13 |
| Melanoma | 7 | 5 | 3 |
| Pancreas | 7 | 6 | 1 |
| Prostate | 6 | 3 | 1 |
