# Supplementary material for: The Expression of Anti-Müllerian Hormone Type II Receptor (AMHRII) in Non-Gynecological Solid Tumors Offers Potential for Broad Therapeutic Intervention in Cancer
Source: Biology (Basel). 2021 Apr 7;10(4):305. doi: 10.3390/biology10040305 (PMC8067808; doi:10.3390/biology10040305)
Supplement: Supplementary file 1 [file biology-10-00305-s001.zip › biology-1127192- Sup Table 3_New.docx]

| Table S3. Clinicopathological features of CRC patients | | |
| --- | --- | --- |
| Patient characteristics | MSS  (N=23) | MSI  (N = 5) |
| Age  Mean (range) | 70 (34-90) | 82 (69-91) |
| Gender  Male  Female | 15  8 | 2  3 |
| Tumor site  Right colon  Left colon  Rectum | 9  11  3 | 4  1  0 |
| Hist. subtype  Mucinous adenocarinoma  Serrated adenocarcinoma  Adenocarcinoma not otherwise specified | 1  0  22 | 0  1  4 |
| pTNM stage  I  II  III  IV | 3  11  6  3 | 0  4  1  0 |
